# Supplementary material for: A Critical View of the Application of the APEX Software (Aqueous Photochemistry of Environmentally-Occurring Xenobiotics) to Predict Photoreaction Kinetics in Surface Freshwaters
Source: Molecules. 2019 Dec 18;25(1):9. doi: 10.3390/molecules25010009 (PMC7017383; doi:10.3390/molecules25010009)
Supplement: Supplementary file 1 [file molecules-25-00009-s001.zip › 3.2.4_gcc-4.4.0.zip!/3.2.4_gcc-4.4.0/3.2.4_gcc-4.4.0/doc/octave/html/interpreter/Operator-Overloading.html]

Operator Overloading - Untitled


Next: Precedence of Objects,
Previous: Function Overloading,
Up: Overloading Objects


---

#### 33.4.2 Operator Overloading

|  |  |  |  |  |
| --- | --- | --- | --- | --- |
|  | Operation | Method | Description |  |
|  | a + b | plus (a, b) | Binary addition |  |
|  | a - b$ | minus (a, b) | Binary subtraction operator |  |
|  | + a$ | uplus (a) | Unary addition operator |  |
|  | - a$ | uminus (a) | Unary subtraction operator |  |
|  | a .\* b$ | times (a, b) | Element-wise multiplication operator |  |
|  | a \* b$ | mtimes (a, b) | Matrix multiplication operator |  |
|  | a ./ b$ | rdivide (a, b) | Element-wise right division operator |  |
|  | a / b$ | mrdivide (a, b) | Matrix right division operator |  |
|  | a .\ b$ | ldivide (a, b) | Element-wise left division operator |  |
|  | a \ b$ | mldivide (a, b) | Matrix left division operator |  |
|  | a .^ b$ | ldivide (a, b) | Element-wise power operator |  |
|  | a ^ b$ | mldivide (a, b) | Matrix power operator |  |
|  | a < b$ | lt (a, b) | Less than operator |  |
|  | a <= b$ | le (a, b) | Less than or equal to operator |  |
|  | a > b$ | gt (a, b) | Greater than operator |  |
|  | a >= b$ | ge (a, b) | Greater than or equal to operator |  |
|  | a == b$ | eq (a, b) | Equal to operator |  |
|  | a != b$ | ne (a, b) | Not equal to operator |  |
|  | a \& b$ | and (a, b) | Logical and operator |  |
|  | a \| b$ | or (a, b) | Logical or operator |  |
|  | ! b$ | not (a) | Logical not operator |  |
|  | a'$ | ctranspose (a) | Complex conjugate transpose operator |  |
|  | a.'$ | transpose (a) | Transpose operator |  |
|  | a : b$ | colon (a, b) | Two element range operator |  |
|  | a : b : c$ | colon (a, b, c) | Three element range operator |  |
|  | [a, b]$ | horzcat (a, b) | Horizontal concatenation operator |  |
|  | [a; b]$ | vertcat (a, b) | Vertical concatenation operator |  |
|  | a(s\_1, \ldots, s\_n)$ | subsref (a, s) | Subscripted reference |  |
|  | a(s\_1, \ldots, s\_n) = b$ | subsasgn (a, s, b) | Subscripted assignment |  |
|  | b (a)$ | subsindex (a) | Convert to zero-based index |  |
|  | display | display (a) | Commandline display function |  |

**Table 33.1: Available overloaded operators and their corresponding class method**

An example `mtimes` method for our polynomial class might look like

```
```
     function y = mtimes (a, b)
       y = polynomial (conv (double(a),double(b)));
     endfunction
```
```
